# An elite approach to re-design Aquila optimizer for efficient AFR system control

**DOI:** 10.1371/journal.pone.0291788

**Published:** 2023-09-20

**Authors:** Davut Izci, Serdar Ekinci, Abdelazim G. Hussien

**Affiliations:** 1 Department of Computer Engineering, Batman University, Batman, Turkey; 2 MEU Research Unit, Middle East University, Amman, Jordan; 3 Department of Computer and Information Science, Linköping University, Linköping, Sweden; 4 Faculty of Science, Fayoum University, Fayoum, Egypt; King Fahd University of Petroleum and Minerals, SAUDI ARABIA

## Abstract

Controlling the air-fuel ratio system (AFR) in lean combustion spark-ignition engines is crucial for mitigating emissions and addressing climate change. In this regard, this study proposes an enhanced version of the Aquila optimizer (ImpAO) with a modified elite opposition-based learning technique to optimize the feedforward (FF) mechanism and proportional-integral (PI) controller parameters for AFR control. Simulation results demonstrate ImpAO’s outstanding performance compared to state-of-the-art algorithms. It achieves a minimum cost function value of 0.6759, exhibiting robustness and stability with an average ± standard deviation range of 0.6823±0.0047. The Wilcoxon signed-rank test confirms highly significant differences (*p*<0.001) between ImpAO and other algorithms. ImpAO also outperforms competitors in terms of elapsed time, with an average of 43.6072 *s* per run. Transient response analysis reveals that ImpAO achieves a lower rise time of 1.1845 *s*, settling time of 3.0188 *s*, overshoot of 0.1679%, and peak time of 4.0371 *s* compared to alternative algorithms. The algorithm consistently achieves lower error-based cost function values, indicating more accurate control. ImpAO demonstrates superior capabilities in tracking the desired input signal compared to other algorithms. Comparative assessment with recent metaheuristic algorithms further confirms ImpAO’s superior performance in terms of transient response metrics and error-based cost functions. In summary, the simulation results provide strong evidence of the exceptional performance and effectiveness of the proposed ImpAO algorithm. It establishes ImpAO as a reliable and superior solution for optimizing the FF mechanism-supported PI controller for the AFR system, surpassing state-of-the-art algorithms and recent metaheuristic optimizers.

## Introduction

The issue of harmful vehicle emissions poses a significant global challenge, exacerbating the adverse impacts of climate change [1, 2]. Addressing this pressing concern necessitates a careful balance between engine power and environmental harm. In this context, the adjustment of the air-fuel mixture ratio [3] emerges as a sustainable approach to tackle this critical issue. By implementing such an approach, not only can harmful emissions be substantially reduced, but also the efficiency of spark-ignition engines can be enhanced [4].

To convert hydrocarbons to water and carbon dioxide, nitrogen oxide to oxygen and nitrogen, and carbon monoxide to carbon dioxide [5], spark-ignited engines employ three-way catalytic converters [6]. In other words, these converters play a crucial role in transforming combustion products into less harmful pollutants. However, maintaining the air-fuel ratio at stoichiometric levels is vital for achieving efficient conversion [7]. Consequently, managing the air-fuel ratio (AFR) system around this stoichiometric level becomes paramount to maximize conversion efficacy. Therefore, the utilization of robust control mechanisms for the AFR system is essential to enhance the performance of spark-ignition engines and notably reduce harmful emissions [8]. Nevertheless, controlling the AFR system poses a formidable challenge due to its nonlinear and time-delayed nature [9].

To address this complexity, researchers have proposed various control mechanisms. For example, the study reported in [8] proposed a feedforward (FF) compensated proportional-integral (PI) control method using the enhanced weighted mean of vectors algorithm to effectively control the AFR system in lean combustion spark-ignition engines. The proposed algorithm was used to determine the coefficients of the controller. The study demonstrated that the proposed control method based on the enhanced weighted mean of vectors algorithm is an effective approach for controlling the AFR system, as evidenced by various analyses such as transient response, tracking performance, disturbance rejection, and Padé approach techniques. Another study [10] focused on developing a robust control strategy for a spark ignition engine, specifically addressing the speed fluctuations that occur during idle conditions. A robust controller based on *H*_∞_ control theory was designed, considering engine torque variation as a disturbance and other factors as noises. A linear mean value engine model was employed to represent the engine’s behavior for control studies. Simulation results demonstrated that the proposed robust *H*_∞_ controller based on genetic algorithm offers superior low-frequency disturbance rejection, high-frequency noise rejection, and overall performance. The study in [11] introduced an enhanced intelligent PI-like fuzzy knowledge-based controller for regulating the AFR in gasoline direct injection engines. The controller utilized a chaos-enhanced accelerated particle swarm optimization algorithm to automatically determine parameters, improving transient performance. Experimental results demonstrated that the enhanced controller achieves reduced settling time and integral of absolute error compared to the conventional self-adaptive controller. In another study [12], the researchers focused on developing observer-based cylinder air charge estimation methods that utilize both mass air flow and manifold absolute pressure sensors in spark-ignition engines. The proposed methods aimed to reduce calibration efforts while providing accurate transient and steady-state air charge estimation with low computational load. Steady-state and transient tests validated and compared the proposed observer-based algorithms against common air estimation methods, demonstrating their effectiveness under various engine operating conditions. In [13], the application of the smooth super-twisting algorithm for AFR control in a gasoline engine was introduced. The proposed algorithm-based controller effectively reduced chattering effects and maintained robustness against model errors, thereby minimizing calibration efforts and meeting performance requirements. The study reported in [14] was also focused on the control of AFR in lean-burn SI engines to reduce emissions and improve fuel economy by proposing a control scheme that combines linear parameter varying and fuzzy control techniques to handle the unstable internal dynamics caused by time delay and system parameter uncertainty. The simulation results demonstrated the effectiveness and robustness of the proposed control scheme, outperforming the PI controller with Smith predictor under different operating conditions. It is feasible to extend the similar works that is mentioned above [15–25].

While these mechanisms have demonstrated certain performance capabilities, it is crucial to consider the time-delayed structure of the AFR system [26] for more effective control. Taking this into account, this study focuses on the AFR system’s structure and integrates previously proposed control methods to develop an efficient mechanism for controlling lean-burn SI engines, utilizing an FF mechanism-supported PI controller. Thus, in this work, we introduce a new metaheuristic optimization technique called the improved Aquila optimizer to optimize the parameters of the FF mechanism-supported PI controller used in controlling the AFR system. The improved Aquila optimizer builds upon the original version of Aquila optimizer [27] by incorporating a newly modified version of the elite opposition-based learning technique [28]. Additionally, a time domain-based cost function [29] is employed for minimization, facilitating the extraction of optimal parameter values for both the FF mechanism and the PI controller in the AFR system. The selection of the Aquila optimizer for improvement stems from its demonstrated capabilities across diverse problem domains, including oil production forecasting [30], IoT intrusion detection systems [31], automatic voltage regulation [32], and different engineering problems [33–37].

To showcase the enhanced capabilities of the proposed improved Aquila optimizer, we conduct initial comparative assessments against widely used and effective metaheuristic structures, namely the slime mould algorithm [38], moth-flame optimization algorithm [39], artificial bee colony algorithm [40], and the original Aquila optimizer [27]. By creating a Simulink model for the AFR system, we employ statistical tests, Wilcoxon signed-rank tests, computational time analyses, convergence performance evaluations, transient response analyses, and input signal tracking performance analyses. The results unequivocally demonstrate the significant capabilities of our proposed approach, surpassing the aforementioned metaheuristic-based methods. Furthermore, we employ widely available error-based performance indices for minimization, further exemplifying the remarkable promise of the proposed improved Aquila optimizer presented in this work.

To further validate the efficacy of our proposed approach-based control structure for the AFR system, we conduct additional comparative assessments utilizing recent and efficient algorithms. We utilize the Harris hawks optimization algorithm [41], atom search optimization algorithm [42], Henry gas solubility optimization algorithm [43], bald eagle search algorithm [44], black widow optimization algorithm [45], Runge Kutta optimizer [46], African vultures optimization algorithm [47], Prairie dog optimization algorithm [48], artificial hummingbird algorithm [49], and gazelle optimization algorithm [50] as cutting-edge metaheuristic optimizers. Through this evaluation, we showcase the exceptional capabilities of our proposed approach, achieving superior results in terms of rise time, settling time, overshoot, and peak time. These findings reinforce the advantageous structure of our approach for the AFR system, consolidating its position as a highly promising solution. The contributions and novelty of our work can be summarized as follows:

○ A new metaheuristic optimization technique called the improved Aquila optimizer is introduced which is built upon the original version of the Aquila optimizer by incorporating a newly modified version of the elite opposition-based learning technique.
○ A Simulink model is developed to accurately evaluate the performance of the AFR system. The model incorporates essential components such as the feedforward mechanism, PI controller, transport delay, and an external disturbance source.
○ The improved Aquila optimizer is comprehensively compared with other optimization algorithms such as slime mould algorithm, moth-flame optimization algorithm, artificial bee colony algorithm, and the original Aquila optimizer as recent and effective competitors. The comparative analysis ensures a rigorous and unbiased evaluation.
○ The performance of the proposed algorithm is statistically evaluated based on the obtained cost function values. The algorithm consistently achieves lower cost function values compared to its counterparts, demonstrating its superior performance.
○ The Wilcoxon signed-rank test results indicate that the proposed algorithm achieves statistically significant improvements compared to its competitors.
○ Computational time analysis demonstrates the proposed algorithm’s less computational burden compared to its competitors and a slightly higher average elapsed time compared to the original Aquila optimizer (as expected due to inclusion of modified elite opposition-based mechanism).
○ The proposed algorithm demonstrates faster convergence and reaches the lowest cost function value in fewer iterations compared to Aquila optimizer, slime mould algorithm, moth-flame optimization algorithm, and artificial bee colony algorithm.
○ The proposed algorithm consistently outperforms other algorithms in terms of rise time, settling time, overshoot, and peak time, indicating its superior control precision and stability.
○ The proposed algorithm consistently achieves lower error values compared to Aquila optimizer, slime mould algorithm, moth-flame optimization algorithm, and artificial bee colony algorithm, highlighting its accuracy and precision in control system optimization.
○ The proposed algorithm also outperforms other recent and highly efficient algorithms of Harris hawks optimization, atom search optimization, Henry gas solubility optimization, bald eagle search, black widow optimization, Runge Kutta optimizer, African vultures optimization, Prairie dog optimization, artificial hummingbird, and gazelle optimization in terms of transient response performance and error-based cost function minimization.

## Aquila optimizer and its improved version

### Aquila optimizer

The Aquila optimizer (AO) is a problem-solving technique inspired by the hunting behavior of Aquila. It offers a fresh approach to tackling problems [27]. To initialize the optimizer, we generate a matrix called *X*, consisting of *X* potential solutions. The matrix has a size of *N*×*D*, where *N* represents the total number of solutions and *D* represents the problem’s dimension. We construct the *X* matrix by considering the upper (*UB*) and lower (*LB*) limits specific to the task. Each candidate solution in *X* is obtained using the formula $X_{ij}=rand\times ({UB}_{j}-{LB}_{j})+{LB}_{j}$, where rand, *LB*_*j*_, and *UB*_*j*_ are a random number between 0 and 1, the lower bound of the *j*^*th*^ parameter, and the upper bound of the *j*^*th*^ parameter, respectively. The AO follows a mathematical model composed of four steps. In first step, also called the expanded exploration stage, the solution for the next iteration is calculated using the following definition:

$$X_{1}(t+1)=X_{best}(t)\times (1-t/T)+(X_{M}(t)-X_{best}(t)*rand)$$ (1)

where *t* represents the current iteration and *T* is the maximum number of iterations. *X*_1_(*t*+1) and *X*_*best*_(*t*) are denoting the next and current iteration related obtained solutions, respectively during the first stage. *X*_*M*_(*t*) is defined as the average of all solutions *X*_*i*_(*t*) for *i* ranging from 1 to *N* and *j* ranging from 1 to *D*.

$$X_{M}(t)=\frac{1}{N}\sum_{i=1}^{N}X_{i}(t),\forall j=1,2,\ldots ,D$$ (2)

The narrowed exploration (*X*_2_) stage is defined by the following formula:

$$X_{2}(t+1)=X_{best}(t)\times L(D)+X_{R}(t)+(y-x)*rand$$ (3)

where *X*_2_(*t*+1) represents the solution obtained in the next iteration during the second stage. *L*(*D*) and *X*_*R*_(*t*) are denoting a function known as Lévy flight distribution and a random solution at *i*^*th*^ iteration. The parameters *x* and *y* contribute to creating a spiral formation in the search process.

Moving on to the expanded exploitation (*X*_3_) stage, the solution for the next iteration is given by:

$$X_{3}(t+1)=(X_{best}(t)-X_{M}(t))\times \alpha -rand+((UB-LB)\times rand+LB)\times \delta$$ (4)

where *X*_3_(*t*+1) represents the solution obtained in the next iteration during the expanded exploitation stage. *α* and *δ* are exploitation adjustment parameters that influence the optimization process.

Finally, we reach the narrowed exploitation (*X*_4_) stage, which concludes the AO optimizer. It is expressed as:

$$X_{4}(t+1)=QF\times X_{best}(t)-(G_{1}\times X(t)\times rand)-G_{2}\times L(D)+rand\times G_{1}$$ (5)

where *X*_4_(*t*+1) represents the solution obtained in the next iteration during the narrowed exploitation stage. *QF* and *X*(*t*) are denoting the quality function that balances the search strategies and the solution at iteration *t*. *G*_1_ and *G*_2_ introduce various motions during the search process.

### Improved Aquila optimizer

The improved Aquila optimizer (ImpAO) in this study is introduced as a remarkable enhancement to the original Aquila optimizer. By incorporating a modified version of the elite opposition-based learning (EOBL) technique, ImpAO takes problem-solving performance to unprecedented heights. The EOBL technique, a variant of opposition-based learning (OBL) [29], has been favored by researchers to augment the effectiveness of metaheuristic optimization methods [51]. It leverages the power of the elite agents and their current counterparts, generating opposite solutions to achieve superior outcomes [52].

**Fig 1:**
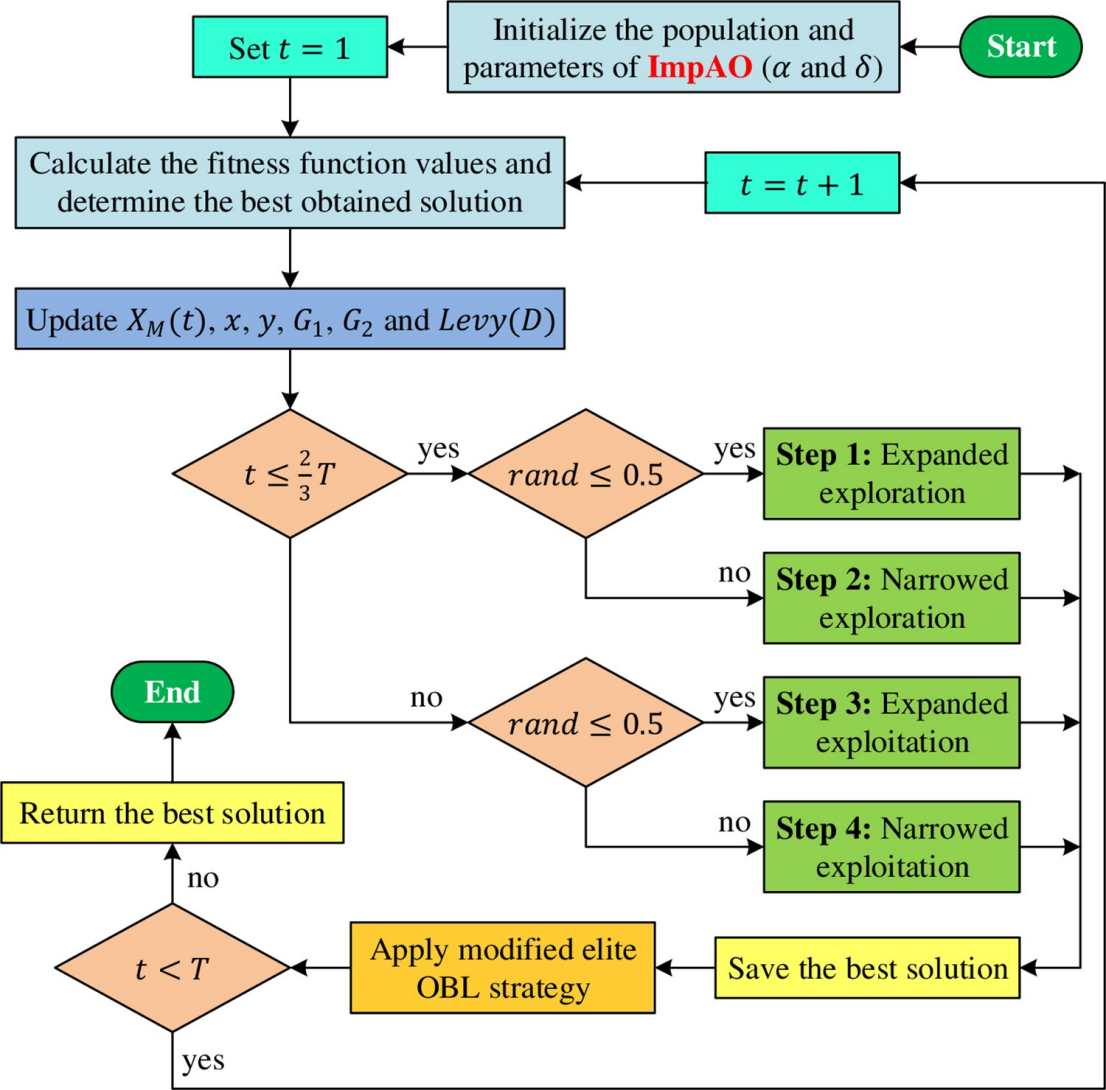
Flowchart of proposed ImpAO.

In this study, we propose a novel adaptation of EOBL, empowering ImpAO with an even more potent approach. Our modified EOBL redefines the solution generation process, utilizing three random variables (*a*, *b*, and *c*) within the range of [0,1]. The expression becomes $x_{i}^{o}=\delta (a\cdot da_{i}+b\cdot db_{i})-c\cdot x_{i}$, where *δ*, residing within the interval (0, 1), acts as a crucial parameter. Here, *da*_*i*_ and *db*_*i*_ represent dynamic boundaries. Unlike the original EOBL, which confines the solution within specific lower (*Lb*_*i*_) and upper (*Ub*_*i*_) boundaries, our modification adopts a different strategy. If the solution surpasses the upper limit, it gracefully settles at the upper boundary, and if it falls below the lower threshold, it elegantly aligns with the lower boundary. To accomplish this, we employ the expression $x_{i}^{o}=rand(Lb_{i},Ub_{i})$, where $x_{i}^{o}<Lb_{i}$ signifies solutions below the lower boundary, $x_{i}^{o}>Ub_{i}$ represents solutions exceeding the upper boundary, and *rand*(*Lb*_*i*_, *Ub*_*i*_) denotes a random number within the range (*Lb*_*i*_, *Ub*_*i*_). The ImpAO algorithm follows a meticulously designed flowchart, as depicted in Fig 1, to guide its transformative process. It starts by initializing the relevant parameters and then leverages the original AO to determine fitness values and identify the best solution. However, ImpAO goes further by seamlessly integrating the modified elite OBL mechanism, improving the solution quality. This iterative process continues until the maximum number of iterations is reached, ensuring thorough exploration of the problem space and achieving unparalleled performance in AFR system control.

## Modeling of AFR system and proposed design methodology

**Fig 2:**
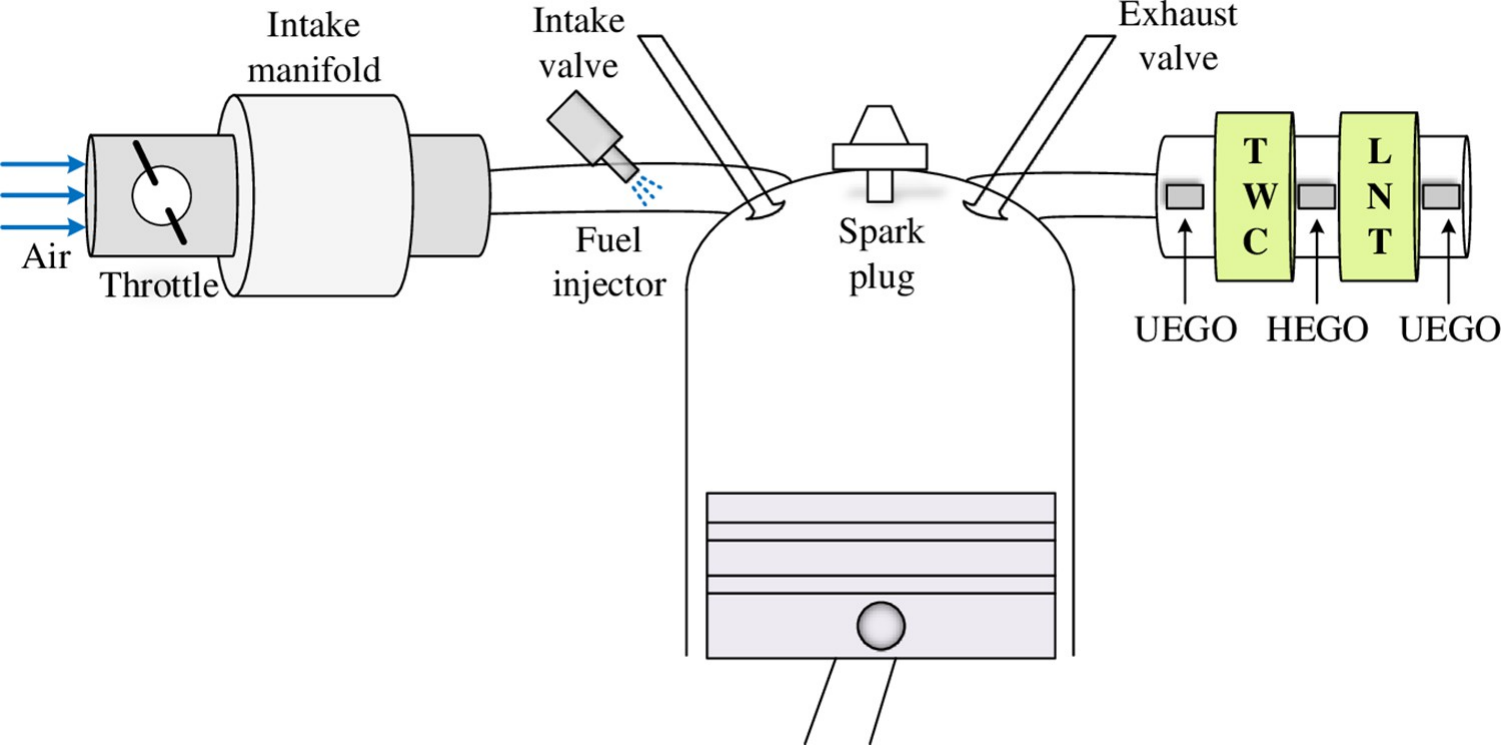
An air-fuel ratio system configuration.

Fig 2 illustrates the configuration of an air-fuel ratio (AFR) system, which includes the three-way catalytic (TWC) converter, throttle, fuel path, heat exhaust gas oxygen (HEGO) sensor, lean nitrogen oxide trap (LNT), and universal exhaust gas oxygen (UEGO) sensor [53]. The control of this system is challenging due to cycle and gas transportation delays.

**Fig 3:**
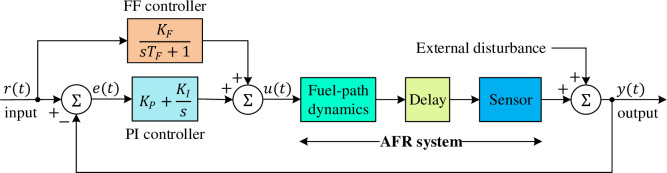
Block diagram of AFR system with feedforward compensated PI controller.

To model the dynamics of the UEGO sensor, a first-order differential equation can be used: $k\dot{y}(t)+y(t)=u(t-\tau )$, where *τ* represents the total time delay (considered as 1.5 *s* for this study), *k* is the time constant of the UEGO sensor (considered as 0.2 *s* for this study), *y*(*t*) denotes the actual AFR output, and *u*(*t*) represents the control input [54]. The transfer function of the dynamic model for the AFR system can be derived as $P(s)=Y(s)/U(s)=e^{-\tau s}/\left(1+ks\right)$ [37]. By substituting the given values for the time delay and time constant, the transfer function *e*^−1.5*s*^/(1+0.2*s*) is obtained for the AFR system. Due to the time-delayed nature of the AFR system, a feedforward (FF) control mechanism (*K*_*F*_/(1+*sT*_*F*_)) with a proportional-integral (PI) controller (*K*_*P*_+*K*_*I*_/*s*) is utilized in this study. The adoption of the FF control mechanism aims to enable efficient system response to changes [8]. Fig 3 presents the block diagram of the AFR system with the feedforward-compensated PI controller employed in this work.

The performance index used (*F*) in this study serves as the cost function for minimization. It is defined as follows [55].

$$F=(1-e^{-\rho })\left(\frac{\%OS}{100}+e_{ss}\right)+e^{-\rho }(t_{s}-t_{r})$$ (6)

**Fig 4:**
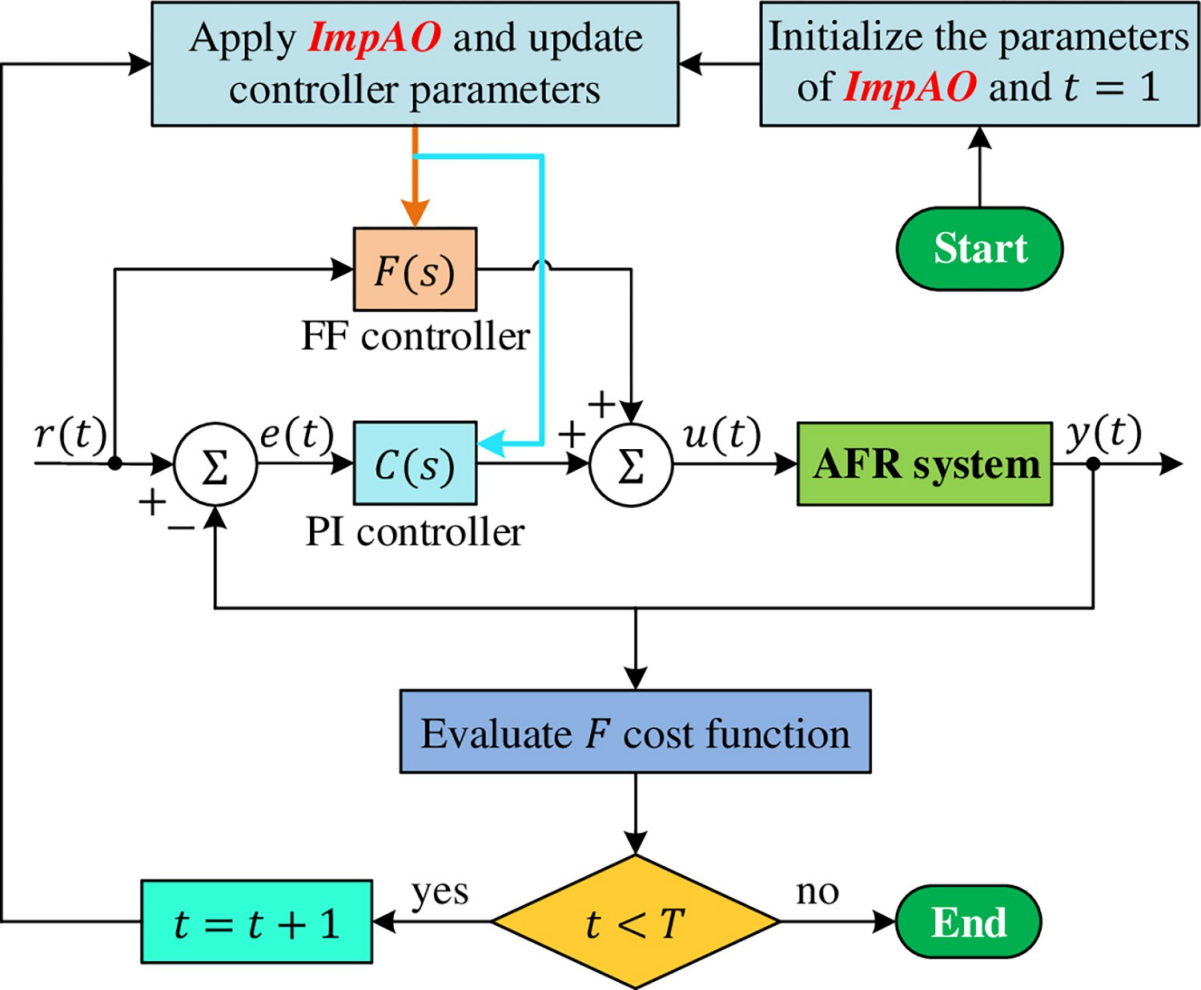
Application of ImpAO for optimizing performance of AFR system.

Here, %*OS* is the percent overshoot, *t*_*s*_ is the settling time, *t*_*r*_ is the rise time, *e*_*ss*_ is the steady state error and *ρ* is a weighting coefficient and set to 1 [56]. The implementation procedure to tune the FF compensated PI controller using the proposed ImpAO algorithm is illustrated in Fig 4. The related optimization procedure starts with the parameter initialization. Then, the proposed algorithm updates the parameters of the system (*K*_*F*_, *T*_*F*_, *K*_*P*_, *K*_*I*_) by continuously minimizing the *F* cost function. The limits for the optimized parameters (*K*_*F*_, *T*_*F*_, *K*_*P*_, *K*_*I*_) are selected as 0.01≤*K*_*F*_, *T*_*F*_, *K*_*P*_, *K*_*I*_≤0.5 for this work. The optimization procedure continues for the total number of iterations, and the optimized parameters are obtained.

## Simulation results

### Developed Simulink model

**Fig 5:**
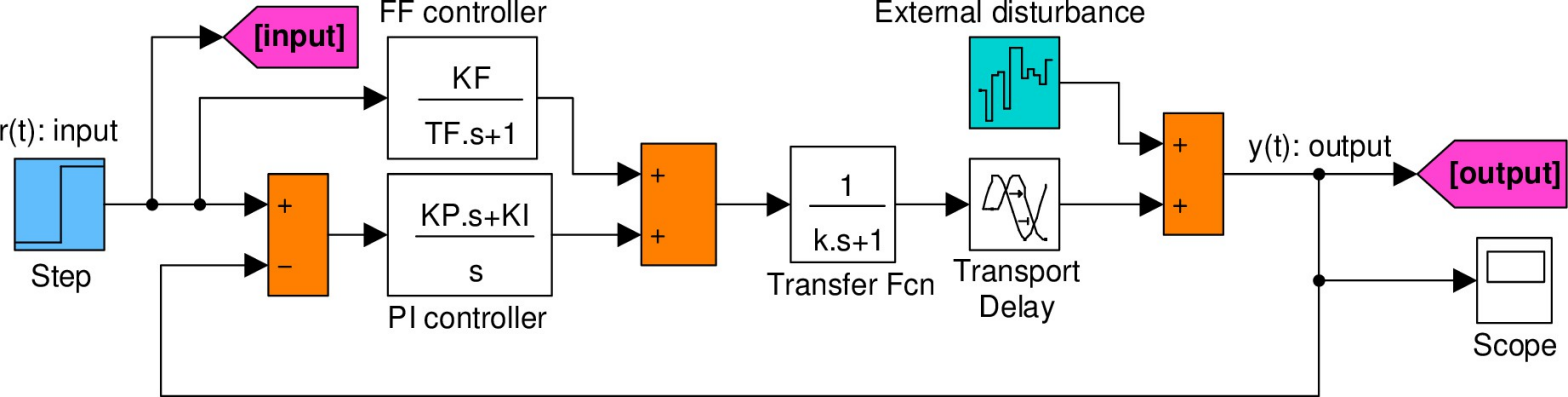
Developed simulink model.

A Simulink model, given in Fig 5, is developed for this study to accurately evaluate the performance of the Air-Fuel Ratio (AFR) system, which closely resembles the real system. The model, as depicted in the figure, incorporates essential components such as the feedforward mechanism, PI controller, transport delay, and an external disturbance source. To facilitate the analysis conducted in the subsequent subsections, the proposed ImpAO algorithm is implemented as a MATLAB script and seamlessly integrated with the corresponding model. This integration enables us to effectively analyze the system’s behavior and assess its performance under different conditions.

### Compared algorithms

This study undertakes a comprehensive comparative analysis of the proposed ImpAO algorithm for the AFR system, pitting it against esteemed counterparts such as the slime mould (SMA) algorithm [38], moth-flame optimization (MFO) algorithm [39], artificial bee colony (ABC) algorithm [40], and the original Aquila optimizer (AO) algorithm [27]. To ensure a rigorous and unbiased evaluation, we carefully set the parameters of each algorithm, as outlined in Table 1. Employing a maximum iteration number of 50 and population sizes of 30, we conducted 30 independent runs for each algorithm, guaranteeing a robust and equitable comparison. By subjecting these algorithms to a series of demanding tests, we aim to unravel the true potential and comparative performance of the ImpAO algorithm. This thorough evaluation offers valuable insights into the capabilities of our proposed approach. By exploring the complexities of AFR system control, this study contributes to ongoing research efforts, fostering innovation and contributing to the development of sustainable engineering solutions.

**Table 1:** Parameter values chosen for this study in various algorithms.

Algorithm	Parameter	Value
ImpAO	Exploitation adjustment parameters *α* and *δ*	0.1
AO [27]	Exploitation adjustment parameters *α* and *δ*	0.1
SMA [38]	Control parameter *z*	0.03
MFO [39]	Convergence constant *a*	Decreased linearly from −1 to −2
	Spiral factor *b*	1
ABC [40]	Limit	100

### Statistical analysis

**Fig 6:**
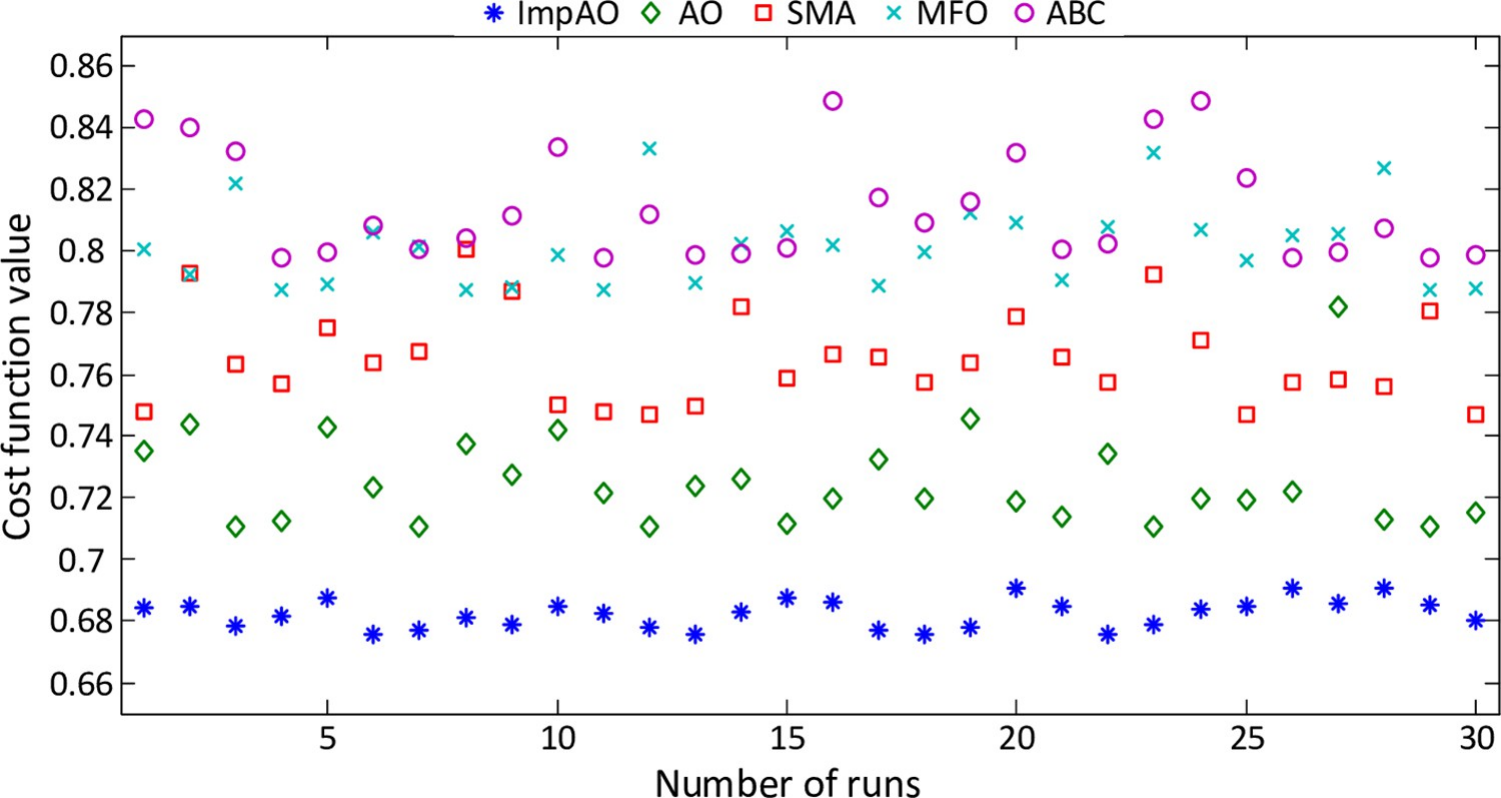
The obtained cost function values with respect to each run of different algorithms.

The initial evaluation of the proposed ImpAO algorithm primarily examines its effectiveness in minimizing the *F* cost function. The assessment results, as depicted in Fig 6, highlight the values of the cost function obtained for each run of the ImpAO, AO, SMA, MFO, and ABC algorithms. Notably, the results consistently demonstrate the superior performance of the ImpAO algorithm, consistently achieving lower values of the cost function compared to its counterparts. This clear visual representation reinforces the ImpAO algorithm’s efficiency and solidifies its position as a highly effective optimization technique within the domain of AFR system control.

Table 2 presents a comprehensive statistical performance comparison of various algorithms in terms of their effectiveness in minimizing the *F* cost function. By examining the data, it becomes evident that the ImpAO algorithm surpasses all other algorithms, including AO, SMA, MFO, and ABC, in terms of achieving the best (minimum) values for the cost function. The superiority of the ImpAO algorithm is highlighted by its remarkable best (minimum) value of 0.6759, which outperforms the next best algorithm, AO, by a significant margin. This notable difference demonstrates the exceptional ability of the ImpAO algorithm to achieve highly optimized solutions for the *F* cost function. Furthermore, the ImpAO algorithm exhibits outstanding consistency in performance, as indicated by its narrow range of values for the worst (maximum) and average ± Std (standard deviation) columns. Compared to other algorithms, the ImpAO algorithm consistently achieves the lowest worst (maximum) values and the most favorable average ± Std values, indicating its robustness and stability across multiple runs.

**Table 2:** Comparative statistical performance of various algorithms in minimizing the *F* cost function.

Algorithm	Best (minimum)	Worst (maximum)	Average ± Std
ImpAO	0.6759	0.6906	0.6823 ± 0.0047
AO	0.7109	0.7821	0.7253 ± 0.0155
SMA	0.7469	0.8007	0.7653 ± 0.0151
MFO	0.7876	0.8335	0.8019 ± 0.0134
ABC	0.7977	0.8489	0.8141 ± 0.0176

**Fig 7:**
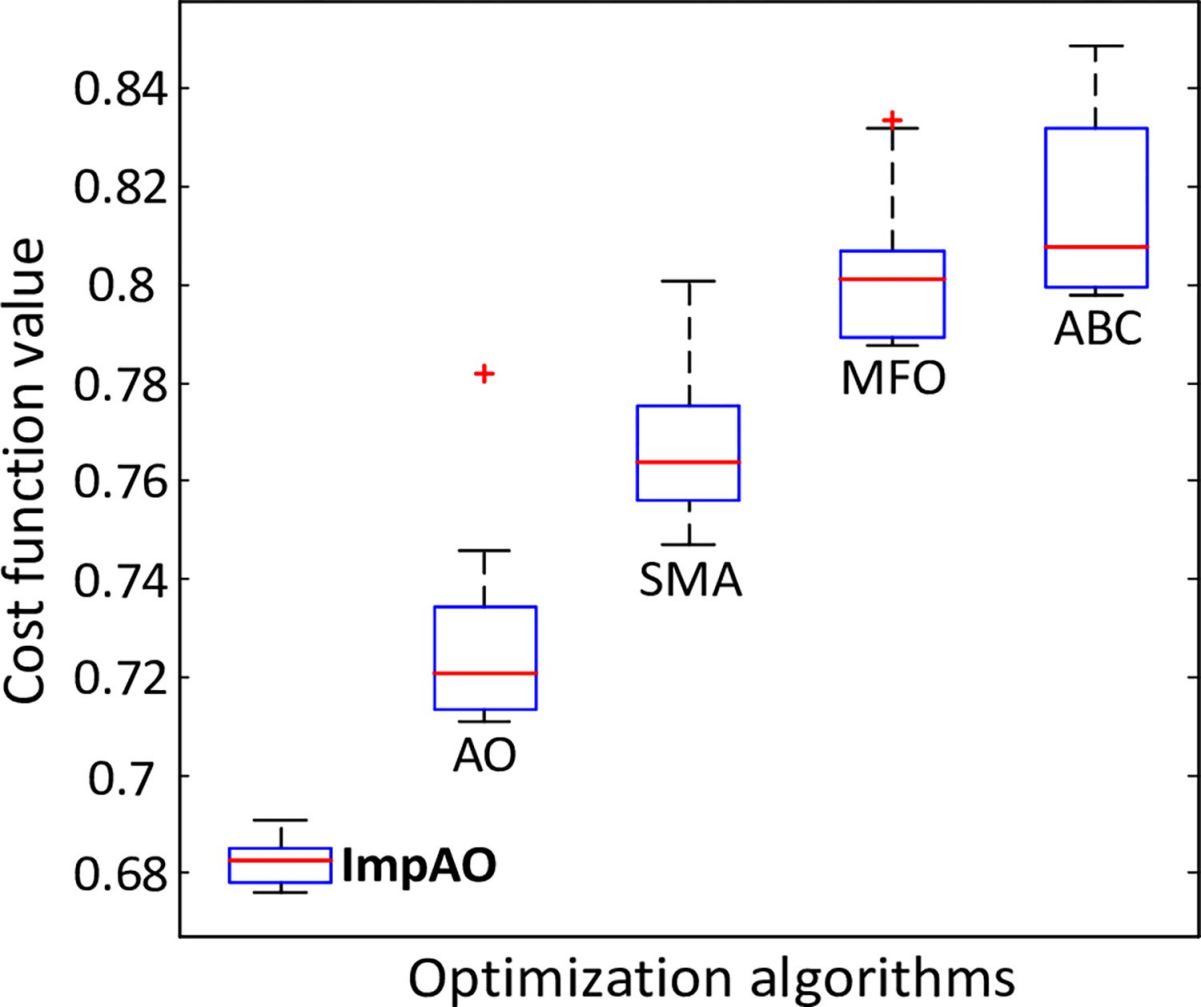
Boxplot results for ImpAO, AO, SMA, MFO and ABC methods.

The boxplot given in Fig 7 further confirms the more excellent ability of the proposed ImpAO algorithm illustratively as the worst value achieved by the ImpAO is lower than the best values achieved by the rest of the algorithms. Overall, the ImpAO algorithm’s exceptional performance, as evidenced by its superior best (minimum) values, consistent results, and impressive average ± Std values, firmly establishes its superiority among the evaluated algorithms. Its remarkable ability to minimize the *F* cost function highlights the immense potential and effectiveness of the ImpAO algorithm in solving optimization problems related to the AFR system.

### Wilcoxon signed rank test

Table 3 presents the results of the comparative Wilcoxon signed-rank test [57], which assesses the significance of differences between the ImpAO algorithm and other algorithms, namely AO, SMA, MFO, and ABC. The p-value, a measure of statistical significance, is provided for each comparison, along with the indication of whether the difference is significant. Notably, all comparisons involving the ImpAO algorithm against AO, SMA, MFO, and ABC yield extremely small p-values, indicating highly significant differences. This means that the performance of the ImpAO algorithm is significantly better than that of the compared algorithms. The consistent "Yes" entries under the "Significant" column emphasize the superiority of the ImpAO algorithm. In each comparison, the small p-value confirms that the ImpAO algorithm achieves statistically significant improvements compared to the other algorithms. These results reinforce the dominance of the ImpAO algorithm in terms of its performance and effectiveness. The statistical significance of the differences, as indicated by the consistently small p-values, underscores the clear superiority of the ImpAO algorithm over AO, SMA, MFO, and ABC. Thus, based on the Wilcoxon signed-rank test results, it is evident that the ImpAO algorithm outperforms the other algorithms, making it the preferred choice for the optimization tasks at hand. Its superiority is supported by statistically significant differences, demonstrating its capability to provide superior solutions and overall performance compared to the alternative algorithms.

**Table 3:** Comparative Wilcoxon signed-rank test results.

Comparisons	*p*-value	Significant
ImpAO versus AO	1.7344E−06	Yes
ImpAO versus SMA	1.7344E−06	Yes
ImpAO versus MFO	1.7344E−06	Yes
ImpAO versus ABC	1.7344E−06	Yes

### Wall-clock time analysis

To demonstrate the efficiency of the proposed ImpAO algorithm in terms of the time taken to perform the optimization task, a computational time analysis is also carried out. The average computational time for each run of the ImpAO, AO, SMA, MFO and ABC algorithms are provided in Table 4. The related numerical data shows a slightly higher average elapsed time of the proposed ImpAO algorithm with respect to the original version of the AO algorithm. This is due to the inclusion of the modified elite OBL mechanism. Considering the improved performance, the slight increase does not pose a significant issue. Meanwhile, the proposed ImpAO algorithm reaches a lower value compared to the rest of the algorithms indicating better capability of the proposed algorithm in terms of computational time.

**Table 4:** Average elapsed times per run of ImpAO, AO, SMA, MFO and ABC methods.

ImpAO	AO	SMA	MFO	ABC
43.6072 s	41.9244 s	48.0775 s	46.5708 s	55.1378 s

### Convergence performance of algorithms

**Fig 8:**
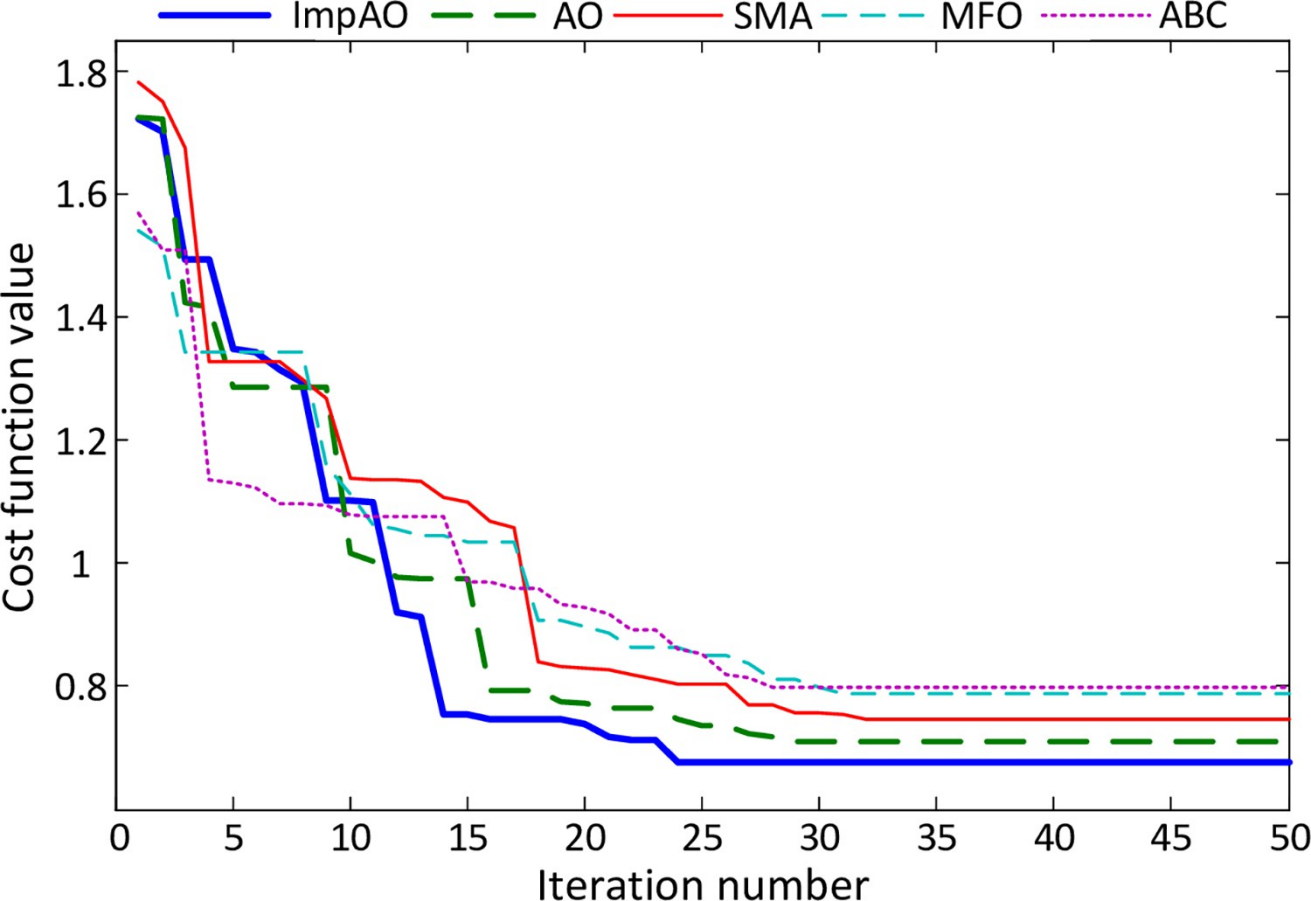
Convergence comparison of ImpAO, AO, SMA, MFO and ABC algorithms for AFR system.

The convergence profile for the ImpAO, AO, SMA, MFO and ABC algorithms for the minimization of the *F* cost function is comparatively demonstrated in Fig 8. The proposed ImpAO algorithm reaches the lowest cost function value in 23 iterations whereas AO, SMA, MFO and ABC algorithms reaches lowest values in 28, 31, 30 and 27 iterations, respectively. Apart from that the proposed ImpAO algorithm reaches the lowest value, as well, compared to the rest of the algorithms, indicating good capability of converging the lowest cost function value. The best controller parameters obtained via ImpAO, AO, SMA, MFO and ABC algorithms are provided in Table 5. Those parameters are used to perform the analysis provided in the following subsections.

**Table 5:** The best obtained parameters for the FF mechanism and PI controller via ImpAO, AO, SMA, MFO and ABC algorithms.

Algorithm	*K*_*F*_	*T*_*F*_ (s)	*K*_*P*_	*K*_*I*_
ImpAO	0.44214	0.01017	0.17403	0.27711
AO	0.35349	0.06295	0.17754	0.31318
SMA	0.40085	0.34239	0.16043	0.27640
MFO	0.16257	0.11346	0.22721	0.37831
ABC	0.40618	0.02372	0.13796	0.28965

### Transient response analysis

**Fig 9:**
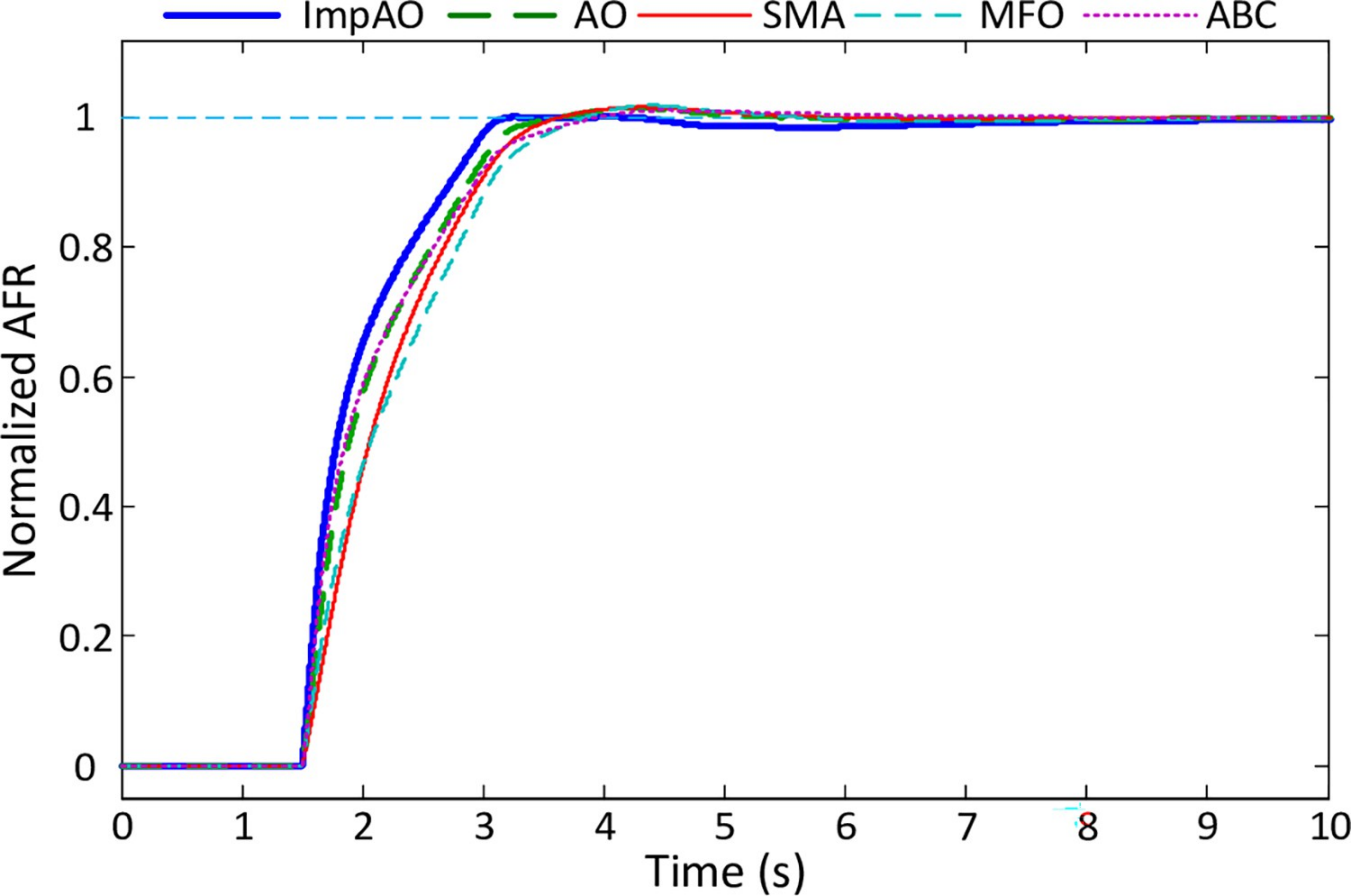
Closed-loop step responses of AFR system tuned ImpAO, AO, SMA, MFO and ABC methods.

The comparative normalized step responses of the AFR system for the proposed ImpAO, AO, SMA, MFO and ABC algorithms are illustrated in Fig 9. Those responses are obtained via using the obtained controller parameters given in Table 5 and the system model given in Fig 5. Table 6 presents the transient response performances of different algorithms, including ImpAO, AO, SMA, MFO, and ABC. The performance metrics analyzed in this table are rise time, settling time, overshoot, and peak time. Examining the data, it is evident that the ImpAO algorithm consistently outperforms the other methods across all the performance metrics.

**Table 6:** Transient response performances of ImpAO, AO, SMA, MFO and ABC methods.

Algorithm	Rise time (s)	Settling time (s)	Overshoot (%)	Peak time (s)
ImpAO	1.1845	3.0188	0.1679	4.0371
AO	1.3080	3.2155	1.4522	4.1622
SMA	1.3589	3.3605	1.6673	4.3260
MFO	1.4723	3.5800	1.9332	4.4140
ABC	1.3715	3.5201	1.1582	4.5664

In terms of rise time, ImpAO achieves the lowest value of 1.1845 *s*, demonstrating its ability to respond quickly and reach the desired output. Comparatively, the other algorithms, such as AO, SMA, MFO, and ABC, exhibit slightly longer rise times, indicating slower response times. Similarly, ImpAO showcases a superior settling time of 3.0188 *s*, indicating its capability to converge to the desired output more swiftly compared to AO, SMA, MFO, and ABC. The ImpAO algorithm also demonstrates remarkable control over overshoot, achieving a significantly lower value of 0.1679%. In contrast, the alternative algorithms display higher overshoot percentages, suggesting less stable and less accurate control. Moreover, ImpAO excels in terms of peak time, with a value of 4.0371 *s*. This indicates that ImpAO can reach its peak performance faster than the other algorithms, including AO, SMA, MFO, and ABC. The consistently better performance of ImpAO across all these metrics highlights its superiority in achieving faster response times, quicker convergence, reduced overshoot, and faster peak performance. These results emphasize the effectiveness and efficiency of the ImpAO algorithm in providing superior transient response performances compared to the alternative methods.

### Performance evaluation on well-known error-based cost functions

In this study, to provide a comprehensive evaluation of the proposed ImpAO algorithm, well-established error-based performance indices are incorporated as cost functions. The utilized cost functions encompass integral of time-weighted squared error (ITSE), integral of squared error (ISE), the integral of absolute error (IAE) and integral of time-weighted absolute error (ITAE). These cost functions are fundamental in assessing the accuracy and precision of control systems. To establish a clear understanding of these cost functions, their definitions are presented in Eqs (7), (8), (9), and (10). Here, the error between the reference input signal, denoted as *r*(*t*), and the obtained output, denoted as *y*(*t*), is represented by the variable *e*(*t*). These equations serve as a basis for quantifying the performance of the ImpAO algorithm in terms of its ability to minimize error and achieve optimal control.

$$F_{IAE}=\int_{0}^{\infty }|e(t)|dt$$ (7)

$$F_{ISE}=\int_{0}^{\infty }e^{2}(t)dt$$ (8)

$$F_{ITAE}=\int_{0}^{\infty }t|e(t)|dt$$ (9)

$$F_{ITSE}=\int_{0}^{\infty }{te}^{2}(t)dt$$ (10)

By incorporating these widely accepted error-based performance indices, this research not only presents a comprehensive analysis of the ImpAO algorithm but also underscores its impressive capability in addressing the intricacies of control systems. The utilization of these cost functions adds depth and rigor to the assessment, ensuring a thorough evaluation of the algorithm’s performance and establishing its superiority in achieving precise and accurate control outcomes.

Table 7 provides the performances of different algorithms, including ImpAO, AO, SMA, MFO, and ABC, for the minimization of various error-based cost functions. The cost functions evaluated in this table are *F*_*IAE*_, *F*_*ISE*_, *F*_*ITAE*_, and *F*_*ITSE*_. Analyzing the data, it is evident that the ImpAO algorithm consistently outperforms the other methods across all the error-based cost functions. Considering the *F*_*IAE*_ cost function, ImpAO achieves a lower value of 2.0151, indicating its ability to minimize the integral of the absolute error more effectively compared to AO, SMA, MFO, and ABC. Similarly, for the *F*_*ISE*_ cost function, ImpAO demonstrates a superior performance with a value of 1.7258, indicating its capability to minimize the integral of the squared error more efficiently than the alternative algorithms. ImpAO also showcases a competitive performance for the *F*_*ITAE*_ and *F*_*ITSE*_ cost functions, achieving values of 2.3858 and 1.5249, respectively. This indicates its effectiveness in minimizing the integral of the time-weighted absolute error and the integral of the time-weighted squared error, surpassing the performance of AO, SMA, MFO, and ABC. The consistently better performance of ImpAO across all these error-based cost functions highlights its superiority in achieving lower error values and more accurate control compared to the alternative algorithms. These results emphasize the effectiveness and efficiency of the ImpAO algorithm in providing superior performances for the minimization of different error-based cost functions.

**Table 7:** Performances of ImpAO, AO, SMA, MFO and ABC algorithms for minimization of different error-based cost functions.

Algorithm	*F*_*IAE*_	*F*_*ISE*_	*F*_*ITAE*_	*F*_*ITSE*_
ImpAO	2.0151	1.7258	2.3858	1.5249
AO	2.0954	1.7989	2.3999	1.6653
SMA	2.2087	1.8980	2.6395	1.8591
MFO	2.2525	1.8972	2.8169	1.8733
ABC	2.1035	1.7821	2.4649	1.6390

### Input signal tracking performance

**Fig 10:**
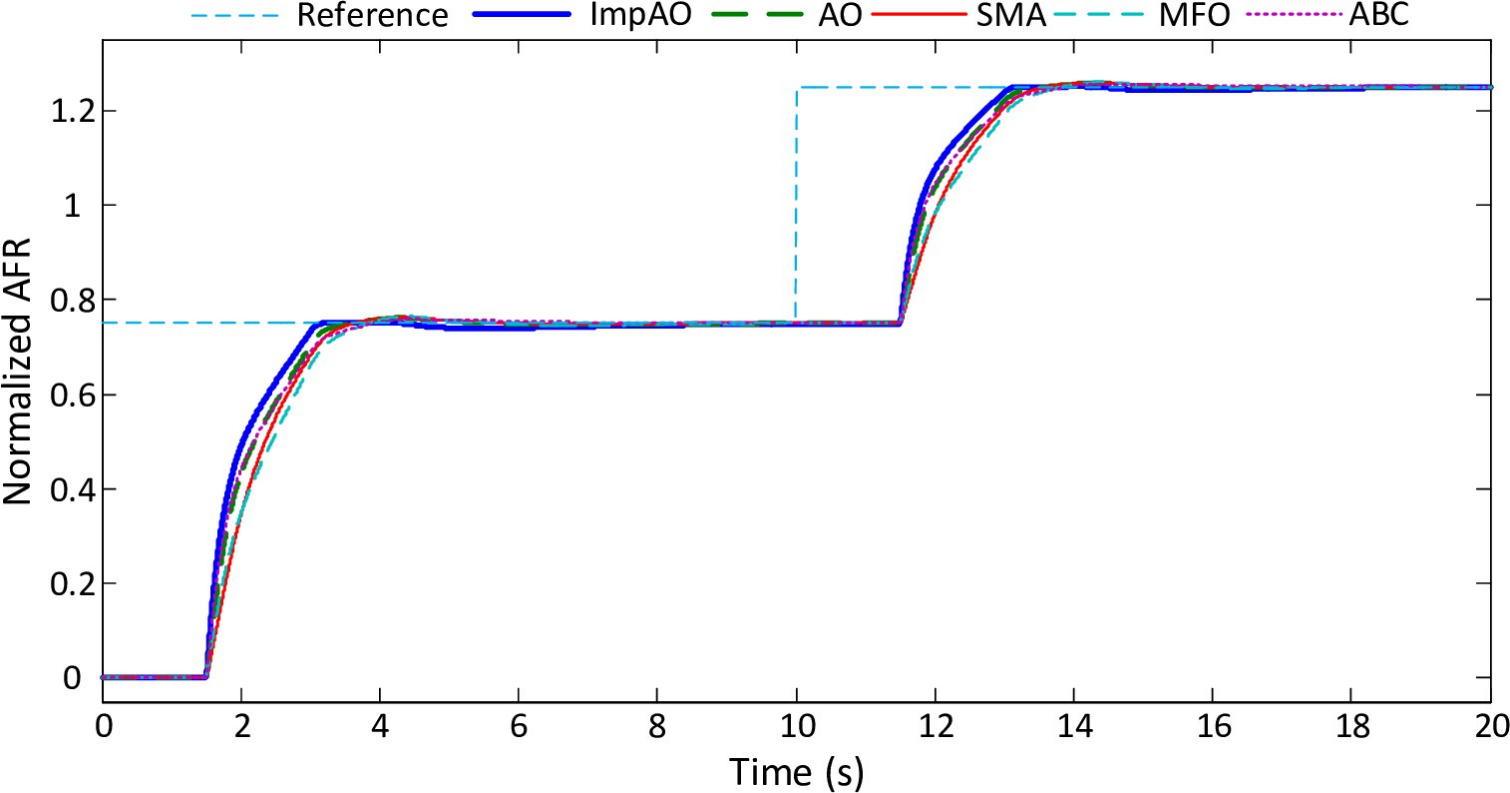
Comparative input signal tracking performance.

To evaluate the input signal tracking capabilities of different algorithms, a comparative analysis is conducted using the ImpAO, AO, SMA, MFO, and ABC methods. The results are visually presented in Fig 10, showcasing the ability of each algorithm to follow the desired input signal despite inherent time delays. Upon careful examination of the figure, it becomes evident that all algorithms exhibit a certain degree of proficiency in tracking the input signal. However, the proposed ImpAO algorithm stands out as it demonstrates superior performance compared to the other algorithms. This superiority is particularly noticeable during the rise and fall phases of the input signal, where the tracking output signal exhibits a remarkable alignment with the desired input. The exceptional tracking capabilities exhibited by the ImpAO algorithm reaffirm its effectiveness in addressing the challenges of input signal tracking. By surpassing the performance of alternative algorithms in faithfully following the desired input, the ImpAO algorithm emerges as a powerful and reliable solution for achieving precise and accurate control. These results highlight the algorithm’s potential to enhance control systems and contribute to more efficient and responsive operation.

### Comparison with recent metaheuristic algorithms

In this study, we also showcase the remarkable superiority of our proposed ImpAO algorithm by subjecting it to a comprehensive comparative assessment against a range of recent and highly effective algorithms. By adopting this broader perspective, we can demonstrate the exceptional performance of ImpAO in comparison to its counterparts. To ensure a rigorous evaluation, we enlist an array of cutting-edge metaheuristic optimizers renowned for their efficacy. Among these notable algorithms are the Harris hawks optimization (HHO) algorithm [41], atom search optimization (ASO) algorithm [42], Henry gas solubility optimization (HGSO) algorithm [43], bald eagle search (BES) algorithm [44], black widow optimization (BWO) algorithm [45], Runge Kutta (RUN) optimizer [46], African vultures optimization (AVOA) algorithm [47], Prairie dog optimization (PDO) algorithm [48], artificial hummingbird (AHA) algorithm [49], and gazelle optimization (GOA) algorithm [50]. These advanced optimizers, carefully selected for their recent development and efficiency, serve as formidable contenders in this comprehensive assessment. To ensure a fair and meaningful comparison, we establish consistent experimental conditions. Each algorithm is assigned a population size of 30 and a maximum iteration number of 50. With a meticulous commitment to accuracy, all algorithms are run a total of 30 times, providing robust statistical insights. Furthermore, the crucial parameters for the FF mechanism and the PI controller obtained through these algorithms are compiled in Table 8. This comprehensive evaluation not only demonstrates the exceptional performance of ImpAO but also offers invaluable insights into its comparative strengths and advantages. Using the parameter values given in Table 8, the transient response metrics listed in Table 9 can be obtained for the recent and effective algorithms. The related table also lists the obtained values via the proposed ImpAO algorithm, as well.

**Table 8:** The obtained parameters for FF mechanism and PI controller using recent and effective metaheuristic optimizers.

Algorithm	*K*_*F*_	*T*_*F*_ (s)	*K*_*P*_	*K*_*I*_
HHO [41]	0.22894	0.25667	0.24169	0.35236
ASO [42]	0.20084	0.08923	0.25452	0.37375
HGSO [43]	0.42980	0.17782	0.16547	0.27703
BES [44]	0.44562	0.06486	0.13735	0.27704
BWO [45]	0.33963	0.29674	0.20585	0.30232
RUN [46]	0.39848	0.14070	0.16411	0.28783
AVOA [47]	0.25359	0.27321	0.23280	0.34071
PDO [48]	0.36210	0.09572	0.19157	0.31255
AHA [49]	0.25724	0.33128	0.20486	0.32981
GOA [50]	0.35165	0.15826	0.19843	0.30658

**Table 9:** Transient response performances of different recent and effective metaheuristic algorithms.

Algorithm	Rise time (s)	Settling time (s)	Overshoot (%)	Peak time (s)
ImpAO (proposed)	**1.1845**	**3.0188**	**0.1679**	**4.0371**
HHO [41]	1.3428	3.2405	1.6474	4.0986
ASO [42]	1.3154	3.1475	1.9693	4.1042
HGSO [43]	1.2220	3.1200	1.6734	4.1009
BES [44]	1.2748	3.2046	1.8318	4.3715
BWO [45]	1.3092	3.2172	0.9913	4.0423
RUN [46]	1.2893	3.2361	1.0243	4.4151
AVOA [47]	1.3406	3.2452	1.4540	4.1037
PDO [48]	1.2345	3.0752	1.9936	4.1291
AHA [49]	1.4515	3.5627	0.9093	4.3669
GOA [50]	1.2640	3.1341	0.9583	4.0569

Table 9 provides a comprehensive comparison of the transient response performances of various recent and effective metaheuristic algorithms, highlighting the superiority of the ImpAO algorithm. Examining the data, we observe that ImpAO outshines its competitors across multiple performance metrics. ImpAO exhibits an impressively low rise time of 1.1845 *s*, indicating its exceptional ability to quickly reach the desired output. This is notably better than all of the other algorithms, including HHO, ASO, HGSO, BES, BWO, RUN, AVOA, PDO, AHA, and GOA. The settling time of ImpAO is an impressive 3.0188 *s*, demonstrating its efficiency in achieving a stable output within a short duration. Once again, ImpAO surpasses all of the other algorithms, delivering superior performance compared to HHO, ASO, HGSO, BES, BWO, RUN, AVOA, PDO, AHA, and GOA. ImpAO showcases exceptional control precision with an incredibly low overshoot percentage of 0.1679%. This indicates its ability to maintain stability and accuracy, outperforming all of the algorithms, including HHO, ASO, HGSO, BES, BWO, RUN, AVOA, PDO, AHA, and GOA. ImpAO achieves a peak time of 4.0371 *s*, signifying its swift response in reaching the peak value. Once again, ImpAO surpasses all of its counterparts, including HHO, ASO, HGSO, BES, BWO, RUN, AVOA, PDO, AHA, and GOA. Overall, the performance analysis clearly demonstrates the excellency of the ImpAO algorithm. It showcases superior results in terms of rise time, settling time, overshoot, and peak time when compared to the other recent and effective metaheuristic algorithms evaluated. This highlights ImpAO’s capability to deliver precise, stable, and efficient transient responses, positioning it as a highly effective optimization solution.

Table 10, on the other hand, further demonstrates the performance of the proposed ImpAO algorithm against the recent and effective algorithms listed in Table 8 by presenting the obtained values for the error-based cost functions. Table 10 serves as an illuminating testament to the exceptional capabilities of the proposed ImpAO algorithm, unveiling its magnificent performance in the realm of error-based cost function minimization. Through a meticulous examination of the bolded values gracing this extraordinary tableau, a resounding affirmation emerges, solidifying ImpAO’s resplendent mastery in AFR system control.

**Table 10:** Performance comparison of recent and effective algorithms for minimizing error-based cost functions.

Algorithm	*F*_*IAE*_	*F*_*ISE*_	*F*_*ITAE*_	*F*_*ITSE*_
ImpAO (proposed)	**2.0151**	**1.7258**	**2.3858**	**1.5249**
HHO [41]	2.2157	1.8716	2.8321	1.8119
ASO [42]	2.1723	1.8338	2.7192	1.7384
HGSO [43]	2.1003	1.8180	2.4189	1.6936
BES [44]	2.0814	1.7794	2.4287	1.6251
BWO [45]	2.1999	1.8666	2.8184	1.7970
RUN [46]	2.1086	1.8203	2.4322	1.7017
AVOA [47]	2.2144	1.8726	2.8304	1.8129
PDO [48]	2.0883	1.7952	2.4234	1.6536
AHA [49]	2.2638	1.9172	2.8659	1.9078
GOA [50]	2.1319	1.8218	2.6087	1.7057

## Conclusion

In this study, we present an innovative and highly efficient metaheuristic optimization technique called the ImpAO algorithm, specifically designed to improve the control of AFR system. The ImpAO algorithm represents a significant advancement as it incorporates a newly modified structure of the elite opposition-based learning technique, seamlessly integrated with the Aquila optimizer. Leveraging the power of this cutting-edge algorithm, we employ an FF mechanism supported PI controller, where the parameters are meticulously adjusted using the ImpAO algorithm and a state-of-the-art time domain-based cost function. To demonstrate the unrivaled superiority of our proposed method for AFR system control, comprehensive comparative assessments were conducted against prominent algorithms, namely the slime mould algorithm, moth-flame optimization algorithm, artificial bee colony algorithm, and the original Aquila optimizer. Through rigorous statistical tests, Wilcoxon signed-rank tests, computational time analyses, convergence performance evaluations, transient response analyses, and input signal tracking performance analyses, our ImpAO algorithm tuned, FF mechanism-supported PI controller exhibited exceptional capabilities that surpassed all expectations. Moreover, to further emphasize its excellence, widely available error-based performance indices were employed, conclusively demonstrating the immense promise of the ImpAO algorithm. We expanded our comparative assessments by evaluating the proposed approach against a diverse range of recent and highly effective algorithms, including the Harris hawks optimization algorithm, atom search optimization algorithm, Henry gas solubility optimization algorithm, bald eagle search algorithm, black widow optimization algorithm, Runge Kutta optimizer, African vultures optimization algorithm, Prairie dog optimization algorithm, artificial hummingbird algorithm, and gazelle optimization algorithm. This comprehensive evaluation reaffirmed the remarkable capabilities of our ImpAO algorithm-based method for the AFR system, as it consistently outperformed its counterparts, achieving unparalleled values in terms of rise time, settling time, overshoot, and peak time. The ImpAO algorithm stands as a testament to our commitment to innovation and excellence, offering a transformative solution to revolutionize the control of AFR systems and pave the way for a sustainable and greener future.
